# Considering the interdependence of clinical performance: implications for assessment and entrustment

**DOI:** 10.1111/medu.13588

**Published:** 2018-04-19

**Authors:** Stefanie S Sebok‐Syer, Saad Chahine, Christopher J Watling, Mark Goldszmidt, Sayra Cristancho, Lorelei Lingard

**Affiliations:** ^1^ Centre for Education Research and Innovation Schulich School of Medicine and Dentistry Western University London Ontario Canada

## Abstract

**Introduction:**

Our ability to assess independent trainee performance is a key element of competency‐based medical education (CBME). In workplace‐based clinical settings, however, the performance of a trainee can be deeply entangled with others on the team. This presents a fundamental challenge, given the need to assess and entrust trainees based on the evolution of their independent clinical performance. The purpose of this study, therefore, was to understand what faculty members and senior postgraduate trainees believe constitutes independent performance in a variety of clinical specialty contexts.

**Methods:**

Following constructivist grounded theory, and using both purposive and theoretical sampling, we conducted individual interviews with 11 clinical teaching faculty members and 10 senior trainees (postgraduate year 4/5) across 12 postgraduate specialties. Constant comparative inductive analysis was conducted. Return of findings was also carried out using one‐to‐one sessions with key informants and public presentations.

**Results:**

Although some independent performances were described, participants spoke mostly about the exceptions to and disclaimers about these, elaborating their sense of the interdependence of trainee performances. Our analysis of these interdependence patterns identified multiple configurations of coupling, with the dominant being coupling of trainee and supervisor performance. We consider how the concept of coupling could advance workplace‐based assessment efforts by supporting models that account for the collective dimensions of clinical performance.

**Conclusion:**

These findings call into question the assumption of independent performance, and offer an important step toward measuring coupled performance. An understanding of coupling can help both to better distinguish independent and interdependent performances, and to consider revising workplace‐based assessment approaches for CBME.

## Abstract 2

The authors put forth coupling as a bridge between the traditional assessment focus on independent performance and the emerging assessment challenge of accounting for collective performance.

## Introduction

Despite an increasing understanding that most clinical practice is not truly ‘independent’, the ability to assess independent postgraduate trainee performance is a key premise of competency‐based medical education (CBME). To achieve this goal, programmes have been required to develop novel competency‐based assessment tools focused on the observation and entrustment of trainees as they complete various tasks.^1^ Characterised as *entrustable professional activities* (EPAs), such tasks are observable and measurable behaviours that supervisors can trust trainees to carry out (*independently*) with success once they have achieved a particular level of competence.^2^ These entrustment decisions are particularly important when it comes to senior trainees preparing for independent practice because they are used to judge their readiness to provide unsupervised patient care.^3^ However, in workplace‐based clinical settings, performance outcomes can be difficult to link to one particular trainee because trainees’ performance is often inseparably tied to the faculty members who supervise them.^4^ Although senior trainees may routinely provide patient care that is neither directly observed nor prospectively approved by faculty supervisors,^5^ even routines such as regular case review interweave faculty members’ and trainees’ clinical decisions and actions.^6^ Furthermore, studies of clinical supervision suggest that faculty members enact various practices that control and even counteract trainee independence to safeguard patient safety, based on factors such as clinical context, patient acuity and trainee experience.^7^ Given that these supervisory practices are sometimes invisible, trainees may perceive more independence than they actually have.^8^

Within medical education, our language has changed from preparing trainees for *independent* practice to preparing them for *unsupervised* or *indirectly supervised* practice, acknowledging that physicians rarely practise in isolation from others. However, this shift has not yet translated into assessment discourse, which retains a strong focus on, and assumption of, independence.^9^, ^10^, ^11^ Given that assessment models are conventionally designed to measure independent performance, they struggle when faced with the collective nature of clinical performance in workplace‐based training environments. In these environments, identifying independent trainee performance for assessment purposes presents a profound challenge.

Assessment research has identified sources of variance such as content specificity,^12^ rater variance,^13^ or the influence of context,^14^ which impact the accuracy of assessments in complex, workplace‐based performance environments. A recent review of rater‐based assessments has also noted inconsistencies that exist between assessment approaches, psychometric assumptions and human capabilities when it comes to assessing trainees in workplace‐based clinical contexts.^15^ Other scholars of workplace‐based assessment have also recognised the challenges of assessing in these environments, with some advocating for more reliance on qualitative data regarding performance,^16^ and others arguing for better training of raters to minimise variance^17^ or more nuanced understanding of the sociocultural influences on practices such as direct observation.^18^ Recognising that many learning activities take place in team settings in the clinical workplace, a variety of approaches have emerged for assessing students’ collaboration competencies.^19^

Although this current scholarship has usefully elaborated the complexity of assessment in clinical and workplace settings, it has tended to maintain the perspective that the independent trainee is the focus of assessment attention. This assumption is pervasive but largely tacit and therefore underexplored. The current study makes explicit, and questions, this fundamental assumption. In doing so we situate ourselves within a rich tradition of sociocultural understandings of clinical learning and practice. Many medical education scholars have argued for the need to move beyond the field's dominant individualist, cognitivist approaches to learning, to engage sociocultural orientations from education, social science and humanities‐based knowledges.^20^, ^21^, ^22^, ^23^ Social learning theories, sociomaterial theories and complexity theory have all been embraced for their ability to foreground entangled dimensions of collaborative work in dynamic clinical systems. Arguments for the value of these orientations highlight their ability: to grapple productively with learning relationships in situated apprenticeships;^20^ to account for both human and material factors involved in workplace practice and learning;^24^ and to trace non‐linear relationships among complex system processes,^21^ including those that may be maladaptive for learning.^22^ Empirical research with these orientations is powerfully advancing our appreciation of issues such as the influences of supervisor interruption during case review,^25^ the entanglement of collaborative practices in clinical teamwork,^26^ the inherent contradictions of simulation‐based education,^27^ and the absence of attention to power in interprofessional education.^28^

Although there has been robust consideration of the sociocultural dimensions of practice and learning, a sociocultural orientation has rarely been applied to issues of *assessment* in medical education. The current study addresses this gap by bridging these two domains and questioning the assumption of independent performance. Such questioning is more than a theoretical exercise. Given the expectation within CBME that trainees will be assessed and judged based on the evolution of their independent clinical performance, we need to better understand under what circumstances their performance is, or is not, independent. The purpose of this study, therefore, was to understand what faculty members and senior postgraduate trainees believe constitutes independent trainee performance in a variety of clinical specialty contexts. Our research question was: Which trainee actions and decisions in the clinical workplace are likely, in the context of CBME, to be considered a reflection of trainees’ independent clinical actions and decisions?

## Methods

This study was approved by the institutional Health Sciences Research Ethics Board. We used a constructivist grounded theory approach to explore the nature of ‘independent’ trainee clinical performances in workplace‐based settings.^29^ Data collection and analysis occurred in an iterative fashion. Using a purposive sampling technique, we conducted individual interviews with 11 clinical teaching faculty members and 10 senior trainees (postgraduate year [PGY]4/5) across 12 postgraduate specialties: anaesthesia, emergency medicine, otolaryngology/head and neck surgery, general surgery, critical care medicine, internal medicine, neurology, obstetrics and gynaecology, orthopaedic surgery, pathology, paediatrics, and psychiatry. We sought a diverse sample because we anticipated that independence would be characterised differently in various specialty programmes, and our aim was to produce a rich description of the features of independent performance that could inform assessment strategies across a variety of programmes making the shift to CBME. E‐mails were sent to faculty members and trainees at a single, midsized Canadian medical school, inviting them to participate in a 30–45‐minute semi‐structured individual interview. We ceased data collection when we reached theoretical sufficiency, which did not mean that no new ideas would have been identified with more data collection, but rather that we had achieved sufficient data collection to enable an understanding of the dimensions of interdependence.^30^, ^31^ During the interview, participants were asked to identify instances of trainees’ independent clinical performance and to describe how such performances are currently documented or captured within the clinical training setting. Participants were probed regarding how the performances they mentioned were influenced by supervisory relationship or team context, two sensitising concepts drawn from the relevant literature. Interviews were recorded and transcribed verbatim and de‐identified prior to data analysis.

We conducted constant comparative inductive analysis using an iterative process in which data collection and data analysis were concurrent, each informing and influencing the other. Following constructivist grounded theory,^29^ the researchers engaged in three analytical stages of coding: initial, focused and theoretical. Initial and focused coding took place iteratively as transcripts became available. Initial coding consisted of reading the interview transcripts line‐by‐line to identify ideas. Building upon our initial codes, focused coding was then used to highlight concepts or themes within the transcripts; an early theme identified was termed ‘the question of decoupling’, which referred to participants’ persistent references to factors influencing the independent performances they were trying to explain. Each new incident, experience or perspective described by a participant was compared with previous incidents, experiences and perspectives to define and refine the theme – the question of decoupling. Our interview guide was also periodically revised in light of this developing analytical process. The iterative nature of collection and analysis also allowed us to use theoretical sampling as the study proceeded, seeking participants from training contexts that might elaborate or challenge our early understanding of this issue of decoupling. Particular attention was paid to these discrepant examples so that our analysis could reflect their occurrence. In regular meetings of the analysis group (SSS, LL and SC), three decoupling subcategories (supervisor, team and system) were discussed and definitions refined, following which the entire dataset was recoded, with careful attention to discrepant instances that challenged the integrity of these thematic categories. At this point in the analysis, we determined that the ‘system’ subcategory was too sparsely populated to be richly described.

Once all thematic categories were finalised and the data organised accordingly, theoretical coding explored the relationships among them, and analytical memos were created to reflect theoretical insights and questions about these relationships. At this point in the process, we shifted our attention from decoupling to coupling, reflecting our emerging understanding that, while participants were explaining why they felt a particular clinical performance was not independent, they were also providing insight into why it was *interdependent*. Insights from theoretical coding were presented to other members of the research team, both individually and in group meetings, for discussion, elaboration and refinement, with special attention to our discrepant examples. Investigator triangulation strengthened the analysis, as our team includes experts in measurement and assessment, teamwork, clinical supervision, postgraduate training and qualitative research.^32^ Our approach also included strategies for returning findings for refinement and elaboration, which serves as a measure of rigour and strengthens our ability to explore our findings’ resonance and evaluate potential transferability to other contexts. There were three venues for this: (i) discussions with four local key informants from specialties outside of those interviewed for this study (i.e. cardiology, reconstructive surgery, radiology and urology); (ii) two local medicine grand‐rounds sessions at different hospitals, where findings were formally presented and participants could ask questions and engage in discussions; and (iii) a public presentation in another province, to an audience of clinical teachers from a variety of specialty contexts. Our discussion of findings with participants and other audiences suggested that the notion of coupling resonated strongly and that it was experienced differently in different specialty cultures and organisational settings.

## Results

The interviews began by asking participants to describe instances of independent trainee performance. Most participants asserted that some clearly independent performances existed in their clinical training context. However, consistent across all interviews, participants spent most of their time detailing an array of disclaimers for, and exceptions to, their examples of independent trainee performance. In analysing these exceptions and disclaimers, we were able to describe patterns of interdependence, which we eventually conceptualised as ‘coupling’. In this results section, we first describe the pattern of responses regarding independent performances, and then we describe two dominant configurations of interdependent or *coupled* performances that we identified in the data. Faculty participants are identified by Participant F# and senior resident trainee participants by Participant R#.

### Independent trainee performance

Participants were able to identify ‘independent’ trainee performances, but there were diverging perspectives in our data about the opportunities to demonstrate independence. Independence was described by our trainee participants (PGY4/5) as occurring when they perform a clinical task, such as gross examination of specimens, without direct supervision (Participant R2), when they provide patient care without prior approval and only consult before disposition or discharge (Participants R5 and R7), or when they are on‐call and make clinical decisions without having to consult with faculty members (Participants R6 and R8). On the one hand, a number of trainee participants characterised ‘everything’ they do as independent. As a senior trainee from emergency medicine explained:

Usually the staff don't see my patients. I'll have done everything, the history and physical, order investigations and even disposition the patient, whether they go home or be admitted to a service. I feel comfortable ordering advanced imaging and CTs, MRIs and my staff trust me to do that as well. (Participant R7)

Most trainees also acknowledged that independent performance was easier to observe in the later stages of postgraduate training (i.e. PGY4/5) and in clinical situations where trainees are working without direct supervision:

So, somebody at my stage. I just finished my time as the chief of the service, so I'm probably as senior as you get. And I would say that at this point I'm very independent. For administrative purposes we'll say, there will always be a staff person in the room or available, but the majority of surgeries I either do on my own or I'm doing with a staff person available to assist as I need them. (Participant R4)

Ordering practices were among the most common examples cited to reflect independent trainee performance. For instance, when asked what sort of independent actions and decisions a trainee might make throughout a shift, one faculty member responded with ‘order lab tests, order medications, and [order] chest x‐rays’ (Participant F1).

However, trainee participants also challenged the very notion of independence within postgraduate training, as a result of their experience that ‘the resident and faculty work as a team’ (Participant R3). Similarly, a faculty participant from critical care commented:

Yeah, the structure of our care delivery is that the residents rarely have the final say on anything, unlike elsewhere in the hospital. We have residents, we have senior residents that we call fellows, and then there is the consultant. So, the residents rarely get to do anything on their own. (Participant F8)

As these comments suggest, trainees work as part of a collaborative team, where performance is interdependent.

### Interdependent trainee performance

As faculty members and trainees described what constituted independent trainee performance, all of them spoke at length about how a performance that seems to be independent may not be. They offered detailed exceptions, disclaimers and complicating factors, such as ‘it's tricky’ (Participant R5), ‘unfortunately’ (Participant R5) and ‘but it is difficult, if not impossible, to separate them by individual practitioner’ (Participant F8), and they appeared to struggle to draw a line around clearly independent performance. For instance, one faculty member from emergency medicine described a complicating factor of trainees being steered toward the sicker patients:

Yes, that [ordering of x‐rays, ultrasound and CTs] is definitely something. Now theirs [trainees] would be skewed, I don't know how you interpret it. (Participant F2)

In analysing these parts of the interviews, we identified recurring patterns of interdependence performance; we have conceptualised these patterns as ‘coupling’ of trainee performance with that of other team members. The main configuration of coupling described by our participants was between trainee and supervisor. Also evident, but less well elaborated, in the data were configurations of coupling between trainees and other members of the interprofessional team.

### Coupling of trainees and supervisors

The predominant configuration of coupling evident in the data was that between a trainee and his or her supervisor. The nature and degree of interdependence of trainee and supervisor depended on the clinical context and the type of supervision provided. In some clinical contexts, such as the operating room, coupling was described in such strong terms that participants questioned whether trainees could ever be considered independent. As one faculty member from obstetrics and gynaecology explained:

I don't believe our residents are given true total independence prior to graduation. Because medicolegally, for example, at the time of a birth if something were to occur, the culture in at least my department in our institution is for the staff person to be available in person at all times. So, you may function independently in the sense of you may put the forceps on and deliver the baby, but not without me actually watching what you're doing still. (Participant F6)

Another surgical faculty member explained that, because of a profound sense of responsibility for the surgical outcome, his level of supervision never abated to allow a trainee full independence:

My patients, their complication rates should be my complication rate, not my residents’ complication rate. I'm watching them like a hawk and if I think they're going to make a mistake we take over. The outcomes of my patients I consider them to be my outcomes, not my residents’ outcomes. (Participant F10)

The clinical context in surgery, where the faculty member is omnipresent in the operating room, supported such careful watching. But this was not unique to surgery. Participants from non‐surgical programmes also reported a sense of being carefully watched. An internal medicine trainee told a story about:

… the busiest night I think I've ever had to work and partly it was because we had a couple of very sick patients and at one point, at 11:00 or so, the staff actually called me and said look it, I was just cruising [the EHR system] and I noticed that you guys have had a lot of consults. Do you have any questions or are you worried about anybody? It was like the most wonderful moment because it was just so comforting to know that somebody was looking over my shoulder someplace. (Participant R6)

Although this trainee expressed relief at knowing he or she was not performing alone but were being watched, others expressed frustration at what they perceived to be a constraint on their independence. The following example comes from pathology and laboratory medicine:

It could be the day before I graduate, and I cannot sign out the simplest specimen. So everything I do still needs to be looked over by a pathologist. It's an odd thing where I graduate the next day and I can sign out everything. So something magical happens that night, and I can suddenly have the ability. It's a challenge in our specialty … Right now, we don't have a lot of independence from that standpoint. Everything we do is, kind of, always looked over. (Participant R2)

Although the ordering of medications, tests or imaging was often mentioned as an independent trainee performance, many faculty participants acknowledged that such orders are often placed only after consultation with a supervisor: ‘for the most part, our residents wait to have the discussion before they actually would order the medication’ (Participant F4). Trainees also commented that affirming clinical orders with one's supervisor before they are placed was a common convention:

This whole very institution, I think you'll find at [our medical school] everything that we do is reviewed with the staff first so that is another layer of I would say complexity that any kind of orders that we put in is very likely to have already been discussed with our staff first in an on call scenario. (Participant R1)

These discussions are rarely evident in documentation, such that ordering practices may appear independent (tagged to the trainee in the electronic health record, for instance) but actually reflect performance that is coupled with the supervisor.

Participants’ responses to the question of independent performance depended in part on their awareness of the work that occurs behind the scenes. One faculty participant from neurology pointed out that some trainees sign‐off on dictations without an awareness that faculty members will review and edit them:

They must come to me. As a consultant, they'll dictate for me. Even if they sign‐off on them, which for the most part we ask them not to so that I can review and edit. But occasionally some residents, if they're off‐service residents, they're used to just signing off on their own dictations. It still comes to me, I have to sign‐off on it, and I can put an addendum. Once it's been finalized by the resident, I can't edit that, but I can put an addendum at the bottom. (Participant F4)

It was not only behind‐the‐scenes actions that coupled trainee performance with supervisors, it was also actions taking place after the trainee's role in patient care had ended. Most trainees admitted knowing very little about the outcomes of their patients, or how the associated clinical documentation for such patients was edited as a result of a faculty member's supervision, because the trainee often did not continue to provide the patient's care:

Sometimes they go straight to the next place. So, that longitudinal course of how that patient did and what happened and how did your decision maybe affect that patient's outcome, we don't often get. We get the immediate, like how they did the next day or within the next week or couple of weeks. (Participant R4)

Faculty members commented that the transient nature of postgraduate training, in which they rotate in and out of clinical workplace settings, makes it difficult for trainees to appreciate how strongly their performance is coupled with their supervisors’, who may have altered clinical decisions:

I change the note, but if … next time I see him, if I see him again, I might mention it, but half of them, I never see them again. (Participant F9)

### Other configurations of coupling

Although coupling between supervisors and trainees was dominant in the data, some participants provided examples of coupling between trainees and other members of the health care team. Across most of the programmes our participants represented, trainees participated as one of many learners on a clinical teaching team. As one trainee explained, the established hierarchy on these teaching teams meant that their performance was intimately connected to the performance of other learners also engaged in patient care activities:

There are two residents actually so usually one senior and one junior. It doesn't matter whether it's senior or junior. The medical student finds whichever is available, and then reviews the case. Dependent on how much work has already been done by the medical student, there may or may not be more work for the resident to follow‐up on so time to disposition is a challenge when you add in the fact that there's a medical student also involved. (Participant R1)

Many participants pointed out that the teaching team structure presents challenges to the attribution of clinical performances, as each trainee's actions or decisions will be coupled, to varying degrees, with a more senior team member with whom they reviewed their plans. And it is not entirely predictable which team members are coupled at any given time: for instance, one trainee reported that ‘anything that I feel needs urgent attention, such as an airway emergency. I would contact both the senior resident as well as the attending’ (Participant R8).

Participants also offered examples of how trainee independence is modulated by the roles and behaviours of team members, such as nurses, social workers and laboratory technicians. One example related to the task of obtaining informed consent:

In an interprofessional practice often it's a social worker who does that [obtain informed consent]. In medicine, it is of necessity needing to be much more interprofessional in its practice. I would hope that our senior residents know how to work with other members of the team. (Participant F11)

As this example suggests, in some institutional contexts, a trainee's practice of obtaining informed consent is likely to be coupled with the social worker's practice. Given that this task is an entrustable professional activity for trainees as they transition to residency, entrustment decisions would need to account for this coupling.

## Discussion

In asking faculty members and trainees to describe instances of *independent* trainee performance, we gained insight into the *interdependence* of trainee performance. We have characterised this phenomenon as ‘coupling’ to capture the interdependence of trainee performance with both supervisors as well as with other trainees and health care professionals. In this section, we situate this conceptual understanding of coupling within the scholarship on clinical supervision and teamwork, and elaborate its implications for workplace‐based assessment. Our aim is to offer the notion of coupling as a conceptual bridge between the traditional assessment focus on independent performance and the emerging assessment challenge of accounting for collective performance in complex clinical environments. We acknowledge that this will be an uneasy marriage of epistemological orientations, but we would contend that such a marriage is necessary to grapple productively with the challenge of assessing coupled performances in clinical training settings.

First, though, a note about the term *coupling*. We have used it to represent interdependence between two team members (e.g. trainee and supervisor) and the term had resonance with participants in our return of findings interviews and presentations. We recognise, however, that coupling is also a term used in organisational science to describe the nature and degree of interdependence of components in complex systems. According to coupling theory, system elements are conceptualised according to their degree of responsiveness (i.e. capacity and ability to respond to changes) and distinctiveness (i.e. preservation of an independent role within a system).^33^ Our use of the term ‘coupling’ in this paper reflects only the most basic of coupling arrangements: between two human elements in a system. However, coupling theory allows for more elaborate relations of interdependence, including among multiple factors, both human and material.^34^ Although the data from this study contained only a few elaborate descriptions of coupling (e.g. coupling between trainee, faculty member and system factors such as rotation schedule or patient census), we expect that as research continues in this domain, we can draw productively on organisational coupling theory to deepen our understanding of the multiple patterns of interdependence shaping trainees’ clinical performance.

It is not surprising that trainee performance is coupled with that of a supervisor. Many studies have highlighted the power and importance of the supervisory relationship in medicine's workplace‐based training model. Hauer and colleagues’^4^ review of clinical supervision identified five important factors that impact trainee independence: supervisor, trainee, supervisor–trainee relationship, task and context. Kennedy's observational and interview study of clinical supervision found that both supervisors and trainees enact strategies to balance the goals of trainee independence and patient safety.^8^, ^35^ Goldszmidt et al.^36^ suggested that supervisors may enable or inhibit trainee independence based on their response to institutional factors such as patient census and discharge pressures. Additionally, Goldszmidt and colleagues argued that the ideal performance of a teaching team is one in which the group comes to an increasingly refined and shared understanding of the patient's needs over the trajectory of the hospital stay, even as individuals rotate on and off the team.^6^ In this way, Goldszmidt's work has shifted the focus from clinical supervision to the intersecting practices of the individuals within the collective team.

Although there is a robust literature on clinical supervision, these studies do not explicitly consider the question of how we conceptualise and assess trainee performance within the context of supervision. Our results extend our understanding of the intersecting practices of supervisors and trainees, and have implications for assessing trainee performance. We have described various configurations of coupled performance between trainees and other members of the clinical teaching team, particularly supervisors. We contend that viewing trainee performance through the lens of coupling can help us in two ways: (i) to identify moments where performance may be truly independent, and (ii) to appreciate when and how performances are interdependent.

### Defining independence

Independence, by definition, means being free from control, influence or support of others, with the ability to think and act for oneself.^37^ As we seek independent performances to assess, we should look for instances in which trainees (i) perform a clinical task without direct supervision (e.g. a lumbar puncture); (ii) gather clinical data without prior approval (e.g. ordering bloodwork); or (iii) manage clinical decision making (e.g. discharge from the emergency room). An appreciation of coupling allows us to clearly articulate such characteristics of independent performance. Once articulated, educators can identify which performances in their own programmes have these characteristics and make strategies for the best way to assess them.

### Characterising interdependence

The concept of coupling we have put forth provides a language for articulating the degree to which a trainee's performance is interdependent with another individual(s). With this language, we can bridge the gap between the assumption of independent performance and the reality of interdependent performance. This gap may explain some of the current challenges in trying to assess trainee performance in authentic, clinical workplace settings. Given that assessment is largely influenced by measurement and psychometrics, obstacles such as the large amount of unexplained variance in workplace‐based assessments or the inability to account for more than one object of measurement in a single observation^15^, ^38^ have received much attention in the literature.

Our work attempts to marry assessment and measurement approaches with an appreciation of sociocultural understandings. We have no expectation that this marriage will be an easy one, as it is likely to challenge sacred assumptions from each of these scholarly communities. However, we would argue that this marriage between assessment and sociocultural perspectives is necessary in order to authentically capture the interdependence of clinical performance in workplace‐based settings. To assess the various configurations of coupled performances, we need approaches that can assess multiple individuals, numerous task dimensions and various outcome measures. We also need to consider how coupled performances could be further characterised by distinguishing between contribution and attribution^39^ in outcomes assessment. Emerging approaches in educational measurement might prove useful to this end. For example, Andrews et al.^40^ recently used the Andersen/Rasch (A/R) multivariate item response theory (IRT) model to assess interaction patterns in dyads and explore how interaction patterns relate to performance outcomes. Using a simulation‐based collaborative problem‐solving task, they found that interaction patterns from two individuals who were previously unacquainted, working to solve a science problem, correlated with performance outcomes. They also characterised the four interaction patterns: cooperative, collaborative, fake collaboration and dominant/dominant that were displayed between the dyads. Wilson and colleagues^41^ have also advocated for the use of models, such as the multi‐level Rasch model, which consider both unidimensional and multidimensional analyses when conducting assessments within collaborative environments. Wilson, Gochyyev and Scalise^41^ used data from partners collaborating in an online learning environment to show that roughly 90% of total variance was explained by groups. These studies from educational measurement provide early approaches for assessing skills such as collaboration in ways that capture aspects of both independent and interdependent dimensions of performance and characterise performance along a spectrum rather than creating a false dichotomy. Within medical education, a variation of the Rasch measurement model proposed by Wilson and colleagues has already been used to capture aspects of rater's collaborative performance.^42^ All of these aforementioned approaches require us to consider the collective in order to meaningfully assess authentic clinical performance.

Our study advocates for a shift towards assessment that (i) is more precise about when trainee performance is independent and (ii) can account for coupling within trainee performance. To illustrate the implications of coupling for assessment, consider the example of trainee ordering practices as an outcome variable. An approach that conceptualises this outcome as coupled would take into account the influence of particular faculty members’ supervisory practices on the extent to which a trainee order is an independent decision or a coupled one. It would also help to determine whether the clinical action could be fairly attributed to a trainee or whether the contribution of the trainee to the final outcome represents a more accurate reflection of the conditions under which the trainee performs. Furthermore, these conditions are not limited to supervisor and trainee; other team members, clinical resources and medical protocols could also contribute to the interdependence of clinical practice. Therefore, assessment approaches would need to incorporate not only data about the trainee, but also data about which faculty member was working with the trainee and where and when they were working, in order to track not only the ordering decision itself, but the supervisory influence and sociocultural conditions surrounding it. Trainees’ ordering practices may well change not only as trainees gain expertise, but also as their supervisors change (and as other salient aspects of the clinical context change from rotation to rotation); an assessment approach designed specifically for coupled performances would allow observation and interpretation of such changes and adjust this information as the configurations of coupling change.

### Limitations

As with any constructivist grounded theory, the findings are a product of the context within which the study was conducted. In our context, the findings are representative of a single medical school; others will need to explore the resonance of coupling in their own settings. Our sampling strategy of interviewing a few supervisors and trainees from a wide range of programmes has allowed us to describe the phenomenon of coupling. However, we cannot yet describe meaningful variations in particular clinical contexts and postgraduate programmes, and purposeful sampling will be required in future studies to pursue such insights. Additionally, our sample included a subset of the many postgraduate programmes in which trainees perform clinical work; as systematic inquiry into coupling continues, we expect the transferability of the concept to be strengthened by examples and exceptions from a wider variety of trainee performance contexts. Given the nature of our data, this paper presents the simplest configuration of interdependence: coupling between dyads. Future research will need to elaborate an increasingly sophisticated description of the configurations of coupling that shape trainee performance in clinical workplaces.

## Conclusion

The concept of collective competence entered medical education discourse in 2008; however, our field has yet to find a meaningful way to translate this into assessment practices. The concept of coupling provides a way forward, as it helps us begin to map the landscape ‘in between’ independent performance and collective performance and to think more purposefully about the constructs we intend to measure. To date, the assessment of independent clinical performance has been fraught with confounding factors such as unexplained variance; we suggest that this ‘noise’ might, in some cases, be a signal of coupling. With this premise, we propose that educators use the concept of coupling to better distinguish independent from interdependent trainee performances so that we begin to develop ways to assess interdependent performances *as interdependent*. If we can develop and employ assessment approaches that more accurately measure trainees’ coupled performance in authentic clinical environments, it will have profound implications for competence judgements in postgraduate training.

## Contributors

SSS contributed to the conceptual design, acquisition, analysis and interpretation, and drafted the work; SC contributed to conceptualisation and analysis, and revised the work; CJW contributed to the conceptualisation and interpretation, and revised the work; MG and SC contributed to the interpretation and revised the work; LL contributed to the conceptual design, analysis and interpretation, and drafted the work. All authors gave final approval of the submitted paper and agree to be accountable for all aspects of the work.

## Funding

(i) Schulich School of Medicine & Dentistry Dean's Research Innovation Grant; (ii) Academic Medical Organization of Southwestern Ontario (AMOSO) Innovation Fund.

## Conflicts of interest

Nothing to disclose.

## Ethical approval

This study was approved by the institutional Health Sciences Research Ethics Board (File Number: 108391).
